# Using Participatory Design Methodologies to Co-Design and Culturally Adapt the Spanish Version of the Mental Health eClinic: Qualitative Study

**DOI:** 10.2196/14127

**Published:** 2019-08-02

**Authors:** Laura Ospina-Pinillos, Tracey Davenport, Antonio Mendoza Diaz, Alvaro Navarro-Mancilla, Elizabeth M Scott, Ian B Hickie

**Affiliations:** 1 Brain and Mind Centre The University of Sydney Sydney, NSW Australia; 2 Department of Psychiatry and Mental Health Pontifical Javeriana University Bogota Colombia; 3 School of Psychiatry Department of Medicine University of New South Wales Sydney, NSW Australia; 4 Neuropsychiatry Research Group Autonomous University of Bucaramanga Bucaramanga Colombia; 5 School of Medicine University of Notre Dame Australia Sydney, NSW Australia

**Keywords:** telemedicine, medical informatics, eHealth, mental health, cultural characteristics, cultural competency, ethnic groups, transients and migrants, quality of health care, international students, Hispanics, Latinos, community-based participatory research, primary health care, patient participation, patient preference, patient satisfaction, consumer health information

## Abstract

**Background:**

The Mental Health eClinic (MHeC) aims to deliver best-practice clinical services to young people experiencing mental health problems by making clinical care accessible, affordable, and available to young people whenever and wherever they need it most. The original MHeC consists of home page with a visible triage system for those requiring urgent help; a online physical and mental health self-report assessment; a results dashboard; a booking and videoconferencing system; and the generation of a personalized well-being plan. Populations who do not speak English and reside in English-speaking countries are less likely to receive mental health care. In Australia, international students have been identified as disadvantaged compared with their peers; have weaker social support networks; and have higher rates of psychological distress. This scenario is acquiring significant relevance as Spanish-speaking migration is rapidly growing in Australia, and the mental health services for culturally and linguistically diverse populations are limited. Having a Spanish version (MHeC-S) of the Mental Health eClinic would greatly benefit these students.

**Objective:**

We used participatory design methodologies with users (young people aged 16-30 years, supportive others, and health professionals) to (1) conduct workshops with users to co-design and culturally adapt the MHeC; (2) inform the development of the MHeC-S alpha prototype; (3) test the usability of the MHeC-S alpha prototype; (4) translate, culturally adapt, and face-validate the MHeC-S self-report assessment; and (5) collect information to inform its beta prototype.

**Methods:**

A research and development cycle included several participatory design phases: co-design workshops; knowledge translation; language translation and cultural adaptation; and rapid prototyping and user testing of the MHeC-S alpha prototype.

**Results:**

We held 2 co-design workshops with 17 users (10 young people, 7 health professionals). A total of 15 participated in the one-on-one user testing sessions (7 young people, 5 health professionals, 3 supportive others). We collected 225 source documents, and thematic analysis resulted in 5 main themes (help-seeking barriers, technology platform, functionality, content, and user interface). A random sample of 106 source documents analyzed by 2 independent raters revealed almost perfect agreement for functionality (kappa=.86; P<.001) and content (kappa=.92; P<.001) and substantial agreement for the user interface (kappa=.785; P<.001). In this random sample, no annotations were coded for help-seeking barriers or the technology platform. Language was identified as the main barrier to getting medical or psychological services, and smartphones were the most-used device to access the internet. Acceptability was adequate for the prototype’s 5 main elements: home page and triage system, self-report assessment, dashboard of results, booking and video visit system, and personalized well-being plan. The data also revealed gaps in the alpha prototype, such as the need for tailored assessment tools and a greater integration with Spanish-speaking services and communities. Spanish-language apps and e-tools, as well as online mental health information, were lacking.

**Conclusions:**

Through a research and development process, we co-designed and culturally adapted, developed and user tested, and evaluated the MHeC-S. By translating and culturally adapting the MHeC to Spanish, we aimed to increase accessibility and availability of e-mental health care in the developing world, and assist vulnerable populations that have migrated to English-speaking countries.

## Introduction

### Background

The need for mental health services far outweighs the capacity of service providers all over the world [1]. Access to adequate-quality mental health care is also limited for many populations but is particularly limited for vulnerable groups such as the elderly and youth populations, racial and ethnic minorities, the socioeconomically disadvantaged, and rural populations [2]. Limited access to services is of particular concern for young people, as it is well established that 75% of the serious mental diseases and substance use problems emerge before 25 years of age [3]. When young people do seek and receive help, timely and evidence-based treatments are encountered by only a small proportion; in some low- and middle-income countries, the treatment gap can be as high as 90% [4].

Populations who do not speak English in English-speaking countries are less likely to receive mental health care [5]. In Latino populations with mental health problems, the lack of English proficiency is one of the biggest barriers when accessing services [6]. In Australia, non–English-speaking migrant populations struggle to access and understand the local health care system [7]. Language proficiency has been identified as a true barrier for migrant men when using services [8].

### International Students

Australia is a popular study destination for students around the world. Most of Australia’s international students are enrolled in the higher education sector (44%), followed by the vocational education and training sector (27%), and English-language intensive courses for overseas students (19%) [9]. Studying abroad can be one of the most remarkable and rewarding experiences, but it can also be a source of great distress. The way migration is experienced by each individual highly depends on the push and pull factors that precipitated the migration [10]. In the case of international students, a high motivation to study in a different country can act as a protective factor, but the cultural distance of the host country, the lack of social support, and academic pressure can be powerful stressors. Consequently, several studies have shown increased rates of mental health problems in this population [10-12].

In Australia, international students have been identified to be disadvantaged compared with their peers; have weaker social support networks; have higher rates of psychological distress [13]; and are at higher risk of experiencing an adjustment disorder or other mental health problems [14]. The “International Student Welfare in Australia” report suggested that Australia does not adequately protect international students’ human rights and highlights mental health as a key area of concern [15]. Recently, awareness of these issues has been covered by Australian mass media due to 27 suicides in the international student population between 2009 and 2015; sadly, all were reportedly associated with low help-seeking behaviors (22%) [16]. As international students are less likely to receive mental health care, the previously mentioned report encourages institutions to provide information, including available services and increased research in this area. However, most of the research has been focused on tertiary education students and none or very little has been dedicated to language students.

This scenario is acquiring a significant relevance in Australia, where the Spanish-speaking (including Latino) international student migration is rapidly growing. In 2016-2017, language student visa grants (subclass 570) increased by 16.8%, where 3 Spanish-speaking countries (Colombia, Spain, and Chile) were situated in the top 10 countries of applications logged outside Australia [17].

### Health Information Technologies

The internet and new and emerging technologies hold enormous promise for significantly expanding the reach of adequate-quality mental health care by addressing several barriers [18]. Interventions delivered through these technologies have the potential to reach a wide geographic area via remote delivery of care [19]; decrease costs in delivering self-help and social networking interventions; and allow for relatively rapid, centralized scaling up of interventions to a public health dissemination level [20]. English-language, Web-based mental health interventions have proven to be effective for self-screening and referral [6], reducing symptoms and delivering effective treatment for major mental health disorders [21]. A large number of studies, including randomized controlled trials, have also demonstrated the effectiveness of various internet-delivered interventions, such as psychotherapy and psychoeducation [22], treating problematic health behaviors [23], and delivering prevention and treatment programs [24]. Other population-based studies have reported that Web-based tools can enhance the delivery of mental health care in primary care settings [25] and support training and supervision for providers [26]. The number of programs available is growing rapidly [27]. Although positive results are seen from the use of self-directed electronic health interventions, increased effectiveness has been reported if they are used as part of a stepped-care model [28], with the support of a trained health professional [29] or as an adjunct to face-to-face treatment [30].

Despite the growth of such technologies in high-income countries, these technologies are still lacking in low- and middle-income countries and, more specifically, in the Spanish language [31]. Traditional telemedicine has supported the cooperation between developed and developing countries to deliver care across borders by linking professionals rather than providing direct connection between professionals and patients [32]. Telepsychiatry has been used to deliver mental health care to individuals requiring attention, not only locally [33-35], but also internationally, as a means to deliver care to Spanish-speaking individuals residing in a different country [32,36,37]. This type of care is a more efficient alternative, as it doesn’t require the use of interpreters and is culturally sensitive [38]. Successful Spanish-language health information technology (HIT) interventions have been applied in several fields, such as cancer; diabetes; and child, infant, or maternal health [39]. Despite this, the HITs available for mental health are scarce. Initial reports have demonstrated their potential utility in the screening of mental health problems [40], as well as in the treatment of depression [31,41,42], anxiety [43], and substance use disorders [44].

Although the development of HITs in Spanish is recent, their usability and retainability among users is of concern [31]. Experience in other languages (mostly English) has demonstrated that participatory design research methodologies that involve stakeholders and end users in the design and development of these systems at all stages could finally increase user engagement and system usability [45-48]. A close collaboration with end users ensures the appropriateness of these systems for culturally and linguistically diverse populations [49]. Therefore, incorporating participatory design research methodologies that puts end users at the center of the design and development process is greatly needed for Spanish-language–based HITs.

### Objectives

The University of Sydney’s Brain and Mind Centre (Sydney, Australia) is a leader in the development of youth-specific mental health services [50,51] and evidence-based electronic health technologies to engage young people in their own care [52]. The Mental Health eClinic (MHeC) [48,53] was a prototypic Web-based tool designed and developed through a partnership between the Young and Well Cooperative Research Centre and the Brain and Mind Centre. The MHeC aimed to deliver best-practice clinical services to young people experiencing mental health problems by making clinical care accessible, affordable, and available to young people whenever and wherever they need it most. The original MHeC had 5 main elements: a home page with a visible triage system for those requiring urgent help; a comprehensive online physical and mental health self-report assessment; a detailed dashboard of results; a booking and videoconferencing system to enable video visits; and the generation of a personalized well-being plan that included links to evidence-based, young person–suggested, health professional–recommended apps and e-tools [53]. We hypothesized that having a Spanish version of the MHeC (MHeC-S) could greatly benefit young people who are native Spanish speakers living in Australia and who are actively seeking help.

Using a research and development cycle (including several participatory design phases) with end users (young people aged 16 to 30 years, supportive others [such as family, friends, caregivers, coaches, teachers, or community members], and health professionals) as a framework, in this study we aimed to (1) conduct co-design workshops with end users to co-design and culturally adapt the MHeC for Spanish-speaking young people based in Australia; (2) inform the development of the alpha prototype of the MHeC-S; (3) test the usability of the alpha prototype of the MHeC-S; (4) translate, culturally adapt, and face-validate the self-report assessment to a Spanish-speaking population based in Australia; and (5) collect information to inform the beta prototype of the MHeC-S.

## Methods

### Participants

Participants included community-based young people aged 16 to 30 years; native Spanish speakers living in Australia; and native Spanish-speaking young people attending headspace Camperdown and headspace Campbelltown (headspace Australia’s National Youth Mental Health Foundation provides early intervention mental health services and assistance in enhancing young peoples’ [aged 12-25 years] well-being; Camperdown and Campbelltown are 2 sociodemographic areas of Sydney, Australia). Additionally, native Spanish-speaking health professionals and supportive others participated. Participants were required to have regular access to a smartphone (with the iOS or Android operating system) and the internet.

The University of Sydney’s Human Research Ethics Committee approved the study (Protocol No. 2014/689 for the co-design workshops and Protocol No. 2016/487 for the user testing sessions). Participants were provided with the relevant information about the study (participant information statement) before providing their consent and participating in the study. We also obtained parental consent for participants under 18 years of age. Young people received gift vouchers to thank them for their time and expertise when they attended the co-design workshops and the user testing sessions.

The recruitment strategy included the identification of potential participants through headspace Camperdown and headspace Campbelltown; poster and postcard advertisements displayed in community organizations; Facebook advertisements and a study-specific Facebook page; use of organizational social media channels; universities, institutes of technical and further education, language schools, and vocational and training institutes; and cooperation with Spanish-speaking consulates in Australia.

### Procedure

**Figure 1:**
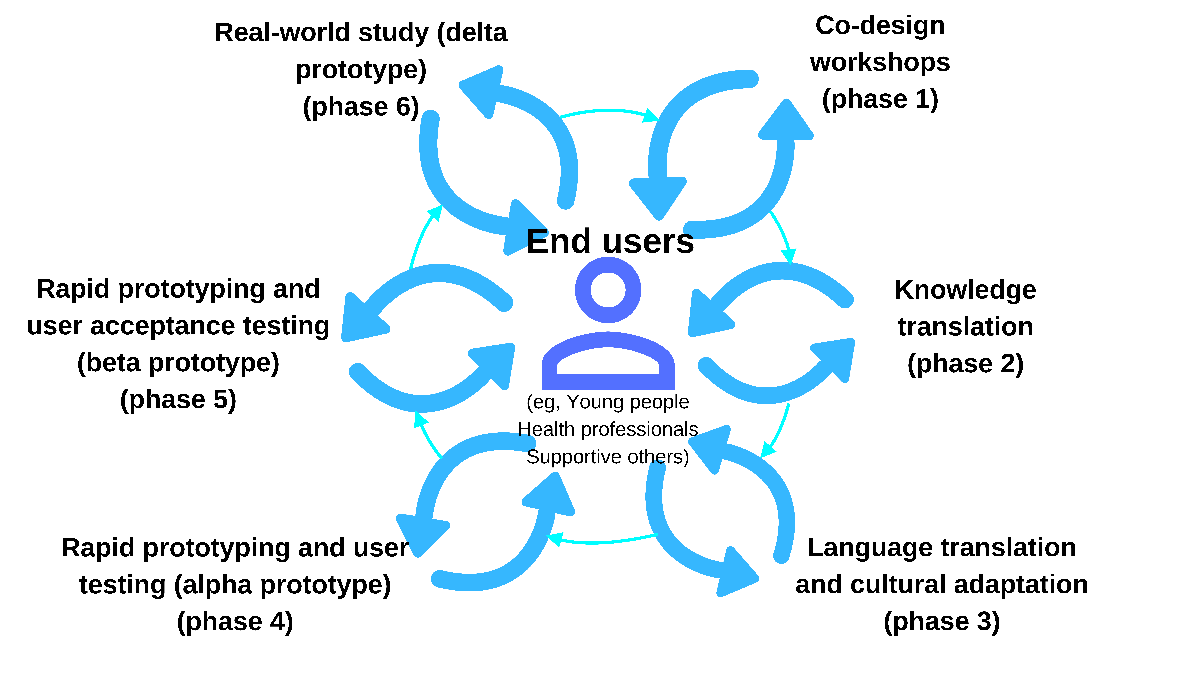
Research and development cycle of the Spanish version of the Mental Health eClinic.

We based the participatory design research methodology on the Young and Well Cooperative Research Centre’s guide *Participatory Design of Evidence-Based Online Youth Mental Health Promotion, Intervention and Treatment* [54]. The research and development cycle used our previously established phases for co-design and build of the original version of the prototypic MHeC [53]. The process encompasses several participatory design phases: co-design workshops (phase 1); knowledge translation (phase 2); language translation and cultural adaptation (phase 3); rapid prototyping of the alpha prototype and user testing (phase 4); rapid prototyping and user (acceptance) testing of the beta prototype (phase 5); and real-world trialing of the final prototype (phase 6). This paper reports the initial 4 phases; we will report phase 5 and phase 6 separately (Figure 1).

### Phase 1: Co-Design Workshops

**Figure 2:**
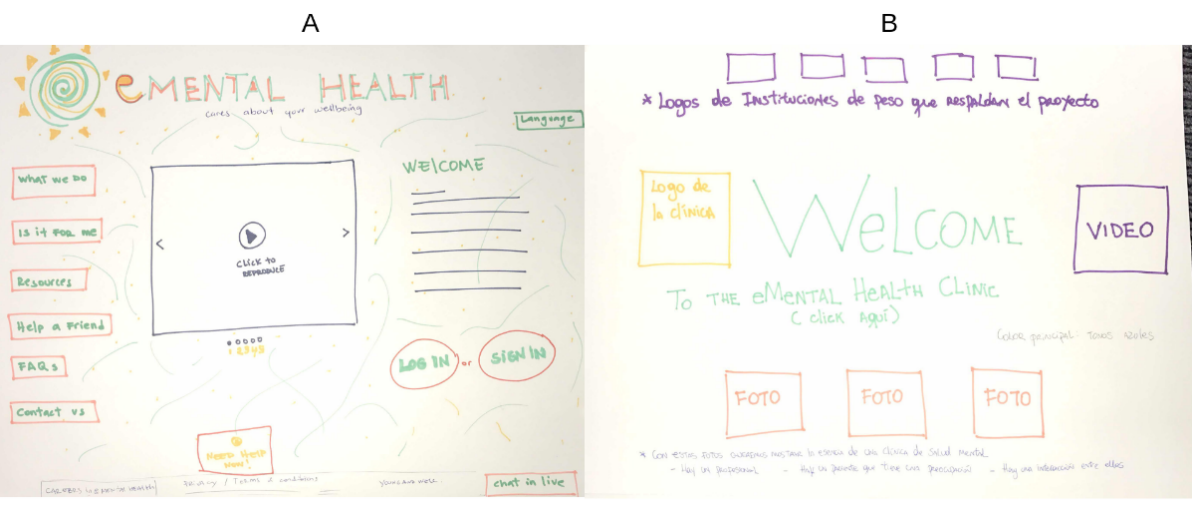
Samples of end user sketches made during a co-design workshop. (A) Hand-drawn sketch by a young person. (B) Hand-drawn sketch by a health professional.

We held co-design workshops in 2 stages with a maximum of 10 participants per workshop; we ran these with young people and health professionals separately. We did not use technology in the workshops; instead, we conducted the following design activities to facilitate the process: we used design testing using mockups and end user sketching (Figure 2) to obtain information for the content, functionality, and the look and feel of the prototype.

The topics covered in each workshop included defining the advantages and disadvantages of having a Spanish version of the MHeC; defining the barriers of having an MHeC-S; assessing the 5 main elements of the MHeC; and defining the functionality and the user interface. At the end of each workshop, the knowledge translation team analyzed and synthetized the information.

### Phase 2: Knowledge Translation

The knowledge translation team independently analyzed the diagrams and notes taken from the previous phase (or workshop), then compared and discussed observations until they reached an agreement. They then synthesized the information by creating wireframes that would be used in the subsequent phase.

### Phase 3: Language Translation and Cultural Adaptation

#### Language of the Prototype

For all of the prototype’s language, a native Spanish-born psychiatrist (LOP) performed a simple translation. Then, for general-content items, 2 Spanish-born psychologists (not involved in the publication of this paper) reviewed the translations. A second Spanish-born psychiatrist (ANM) also reviewed specific mental health content or sensitive content such as the dashboard of results and psychoeducational factsheets. Discrepancies between the translations were resolved by consensus in the group.

#### Translation and Cultural Adaptation of the Self-Report Assessment

Understanding the great need for health professionals and researchers to have available, reliable, and valid measures across different languages and populations, we aimed to translate, back-translate, culturally adapt, and face-validate the Australian self-report assessment using a modified version of the “user-friendly guideline for the translation, adaptation and validation of instruments or scales for cross-cultural health care research” developed by Sousa and Rojjanasrirat [55].

Two Spanish bilingual health professionals (LOP and ANM) independently translated all (health-related) items from English to colloquial Spanish, with the exception of standardized surveys already available in Spanish. A third native Spanish-born psychologist (AMD) reviewed the translated versions and the original English versions. Then, in group discussions, we ensured that all items were linguistically and culturally appropriate by assessing the face-validity of each item in the self-report assessment, as well as assessing the readability and grammatical consistency of the entire assessment. All items were then back-translated to English by one Australian adult (not involved in the publication of this paper) who is fluent in colloquial Spanish and is based in Colombia, has extensive research experience and tertiary qualifications in health, and is accredited to teach English to adults. Discrepancies between the original and back-translated versions were resolved in group sessions between the translators and back-translator.

A literature review was undertaken (by LOP) to identify relevant measures for this population, as well as those instruments already translated and validated into Spanish. The review included both published (identified via PubMed, Google Scholar, SciELO, and LILACS) and gray literature (identified via Google Advanced search) in both English and Spanish. Understanding that some questionnaires might have several translations or versions, we established the following process to select the instruments: first, we selected official translations; if these were unavailable, we selected versions of the published translation and psychometric processes. When more than 1 version or source was available, the 2 previously mentioned health professionals (LOP and ANM) by consensus selected the most appropriate to be included.

### Phase 4: Rapid Prototyping and Usability Testing of the Alpha Prototype

Phase 4 involved user testing with end users: young people, health professionals, and supportive others. Sessions used laptops, tablets, and smartphones (with the iOS or Android operating system), where participants had access to the alpha prototype of the MHeC-S. In each 90-minute one-on-one user testing session, a researcher was paired with an end user. Using a think-aloud protocol [56], participants were observed as they navigated the prototype and responses were recorded to questions posed by the researcher about the main elements of the MHeC-S. A total of 4 usability tasks were completed in the session: (1) create an account and log in, (2) find the Need Help Now button, (3) explore the dashboard of results, and (4) book an appointment. Task completion (yes/no) and task difficulty were measured using the Single Ease Question (SEQ; responses ranged from “very difficult” to “very easy,” rated from 1 to 7) [57]. User testing also explored the prototype’s utility and the users’ inclination to use the MHeC-S, overall comments, and naming of the prototype. Interviews and observations were transcribed. No instructions or clues were provided, and all responses and observations (eg, nonverbal cues) were transcribed.

### Data Analysis

We simultaneously collected and analyzed data at the end of each phase in order to facilitate the process. Hence, we explored preliminary findings in the following phase. We determined interrater reliability and analyzed task difficulty using IBM SPSS Statistics for Mac 22.0 (IBM Corporation). We uploaded and analyzed source documents (workshop discussion notes, artifacts [mockups and end user sketches] and user testing notes) using thematic analysis techniques [58] in NVivo 11 for Mac (QSR International) [59]. The thematic analysis framework involved both inductive and deductive coding. Acknowledging that one of the biggest challenges in this project was the translation and cultural adaptation of the MHeC-S, in the deductive code framework we considered the adaptation of the prototype in 4 dimensions: technology platform, functionality, content, and user interface. As defined by Valdez et al in their culturally informed design framework [60], “technology platform” refers to the different types of hardware, “functionality” indicates the actions that can be performed, “content” refers to the message that is transmitted, and “user interface (design)” refers to the visual presentation of the content and functionality. We also enriched this type of coding with our previously established [53] codes (general elements of the MHeC; general look and feel; privacy and data sharing; and interaction of the MHeC with social networks).

Data collection and qualitative analysis were done in Spanish. To facilitate reporting, we provide quotes translated from the original data. Multimedia Appendix 1 shows the original quotes in Spanish.

One researcher coded all the material (coder A: LOP) and a second coder (coder B: not involved in the publication of this paper) reviewed half of the collected documents in order to assess the reliability, assess consistency, and reduce potential bias [61]. We calculated interrater agreement for each theme using the Cohen kappa statistic on a binomial distribution (category present vs category not present) for each of the themes [62] and interpreted the obtained values using Viera and Garrett’s criteria: kappa range .01 to .20 indicates slight agreement, kappa range .21 to .40 indicates fair agreement, kappa range .41 to .60 indicates moderate agreement, kappa range .61 to .80 indicates substantial agreement, and kappa range .81 to .99 indicates almost perfect agreement [63].

## Results

### Workshops and Sessions

In May 2015, we conducted 1 full-day co-design workshop with Spanish-speaking young people based in Australia and 1 full-day co-design workshop with Spanish-speaking health professionals based in Australia. In total, we conducted 3 knowledge translation sessions immediately after each workshop and at the end of the usability testing. The general-content translation process started in June 2015, and the self-report assessment literature review and translation process started in January 2016 and lasted until the end of the same year. We conducted 15 one-on-one user testing sessions between March and November 2017.

### Participant Characteristics

A total of 10 young people participated in the co-design workshops; 8 were female and their ages ranged from 17 to 29 years (median age 24 years). Of the young participants, 8 were Colombian and 2 were Chilean. A total of 7 health professionals participated in the workshops; 6 were female and their ages ranged from 22 to 34 years (median age 28 years). Of the health professionals, 3 were from Colombia, 2 were from Chile, and 2 were from Spain.

A total of 15 participants participated in the one-on-one user testing sessions: 7 young people with ages ranging from 19 to 30 years (median age 26 years); 5 health professionals with ages ranging from 27 to 74 years (median age 35 years); and 3 supportive others with ages ranging from 30 to 57 years (median age 30 years). Of these participants, 10 were female and 12 were Colombian, while the rest were from Argentina, Spain, and Venezuela.

### Thematic Analysis

We collected and analyzed a total of 225 source documents (2 workshop discussion notes and 208 artifacts produced by participants were collected in the co-design workshops plus 15 user testing notes) during the entire process.

#### Coding Interrater Reliability

Using inductive coding, 1 new main theme emerged (help-seeking barriers) and, from the deductive coding framework, 4 main themes were reiterated (technology platform, functionality, content, and user interface). Of the 225 source documents, 106 (47.1%) were analyzed by both raters. A total of 378 annotations were recoded from both coders (coder A and coder B). Interrater agreement of functionality theme between coder A and coder B was “almost perfect” (kappa=.86; *P*<.001), with concordance in a total of 93.7% (354/378) of the annotations. Similarly, we obtained an “almost perfect” agreement (kappa=.92; *P*<.001) between raters in relation to the content theme, with 97.6% (369/378) of total concordance. In relation to interface, interrater agreement between coders was “substantial” (kappa=.785; *P*<.001), with concordance in a total of 90.0% (340/378) of the annotations. In this random sample, no annotations were coded for help-seeking barriers or technology platform themes.

#### Help-Seeking Barriers

Within this domain, participant perceptions of the help-seeking barriers fell into 3 categories: the language barrier, problems recognizing symptoms or poor mental health literacy, and the availability and accessibility of sources of help.

##### Language Barrier

All participants (32/32, 100%) highlighted language as the main barrier to getting medical or psychological services (Multimedia Appendix 1):

...even if I needed to call 000, I wouldn’t be sure if they understand what I’m saying...

Young person, quote A

I don’t think that I would be able to explain my feelings to someone in English...

Young person, quote B

As the aim for most of these students was to learn English (or improve their English level), their communication skills were, in general, limited. This was a source of distress, as they felt limited in their day-to-day living:

...it’s very hard to arrive here (Australia) and not understand what is happening...

Young person, quote C

...understanding simple instructions—like where is the train stop—is very difficult...

Young person, quote D

For some, the language barrier could have a very negative impact on their confidence:

...it’s like in English I’m a different person; sometimes I feel people think I’m dumb...

Young person, quote E

...the impact on their [international students] confidence is huge. Sometimes I have to remind him [international student] what he is capable of...

Health professional, quote F

##### Problems Recognizing Symptoms or Poor Mental Health Literacy

International students face different issues during migration that could have an impact on their well-being. Participants felt concerned for those who have recently arrived in Australia, as they are perceived as being more vulnerable. According to these participants, a great majority experienced some degree of cultural shock upon their arrival; getting used to regular things such as food, climate, and transport can be relevant stressors among students.

...it is hard to try to fit, and try to understand how things work here...

Young person, quote G

As Australia’s cost of living is high, all participants reported economic concerns (32/32, 100%), whereas some (17/32, 53%) experienced difficulties with housing, getting a job (or a job with fair work conditions), or establishing relationships with peers. All these factors put the students at a higher risk of adaptational problems, which are often unnoticed.

Additionally, the conditions of migration greatly affect individuals’ experience in a new country. Some common negative factors were visiting another country for the first time, travelling alone, and not having family members or friends already residing in that country. Most international students need to work to pay their expenses; however, the jobs they find to support their stay are often not related to their already acquired skills, as a young person explained:

...the majority [of] us have Bachelor degrees in our home countries...so when we arrive in Australia, the jobs we find are very different from what we have been trained in—most of us have to work cleaning, or as a waitress or in construction

Young person, quote H

For many young people, reconciling this discrepancy is challenging.

##### Availability and Accessibility of Sources of Help

All 12 health professionals and all 3 supportive others believed that international students have a great need for Spanish-language–based mental health services in Australia. They perceived that the cases of young people requiring help are increasing, as a supportive other explained:

...possibly one international student dies by suicide every year here, and more frequently we have to provide assistance to students that are hospitalized for a mental health concern...

Supportive other, quote I

Young people believed that having an MHeC-S would be of great utility, as they struggle to understand Australia’s health system and are not aware of their Overseas Student Health Cover benefits. All young participants (17/17, 100%) knew Australia’s national emergency phone number (000). However, just a few (7/17, 41%) of them understood where to go if they needed nonurgent medical care, and all of them stated that they didn’t know where to get psychological assistance.

All 17 young people said they would use a system like the MHeC-S, as they felt it would be a tool to increase mental health awareness and access to sources of help. Additionally, students believed they would be more inclined to use the MHeC-S if they knew about it beforehand, perhaps in the information they receive before arriving in Australia. All 12 health professionals imagined the prototype acting as a bridge between established services and centers in Australia such as the Transcultural Mental Health Centre; New South Wales (NSW) Service for the Treatment and Rehabilitation of Torture and Trauma Survivors; Translating and Interpreting Service; NSW Spanish and Latin American Association for Social Assistance; other relevant cultural associations; and diplomatic missions.

In relation to online sources of help, participants stated that Google was their main source for getting information about their health symptoms. However, they did not necessarily trust all the information they obtained. Participants agreed that there is a shortage of Spanish-language online information (from reputable sources such as universities and organizations) and, more specifically, trustworthy apps and e-tools.

#### Technology Platform

All participants (32/32, 100%) reported that they had constant access to the internet via mobile data plans or Wi-Fi networks. The most commonly used device to access the internet was a smartphone (32/32, 100%), followed by a laptop. All participants agreed that the MHeC-S needs to be accessible via a mobile device in order to really respond to this population’s needs, as some of the students did not have a desktop, laptop, or tablet. All 17 young people reported that mobile phones and the internet were necessary tools in this period of their life, as they used them to communicate with English-speaking people and keep in contact with family and friends overseas. Additionally, they used them in their daily activities (eg, a global positioning system feature), or as a way to find a job or accommodation. As a consequence, they highlighted the importance of the MHeC-S having a responsive Web design, where the prototype needs to work on mobile devices; otherwise, access would be jeopardized.

#### Functionality

There was adequate acceptability of the 5 main elements of the MHeC-S: a home page with a visible triage system for those requiring urgent help; a comprehensive online physical and mental health self-report assessment; a detailed dashboard of results; a booking and videoconferencing system to enable video visits; and the generation of a personalized well-being plan that includes links to evidence-based, young person–suggested, health professional–recommended apps and e-tools.

##### Element 1: Home Page and Triage System

**Figure 3:**
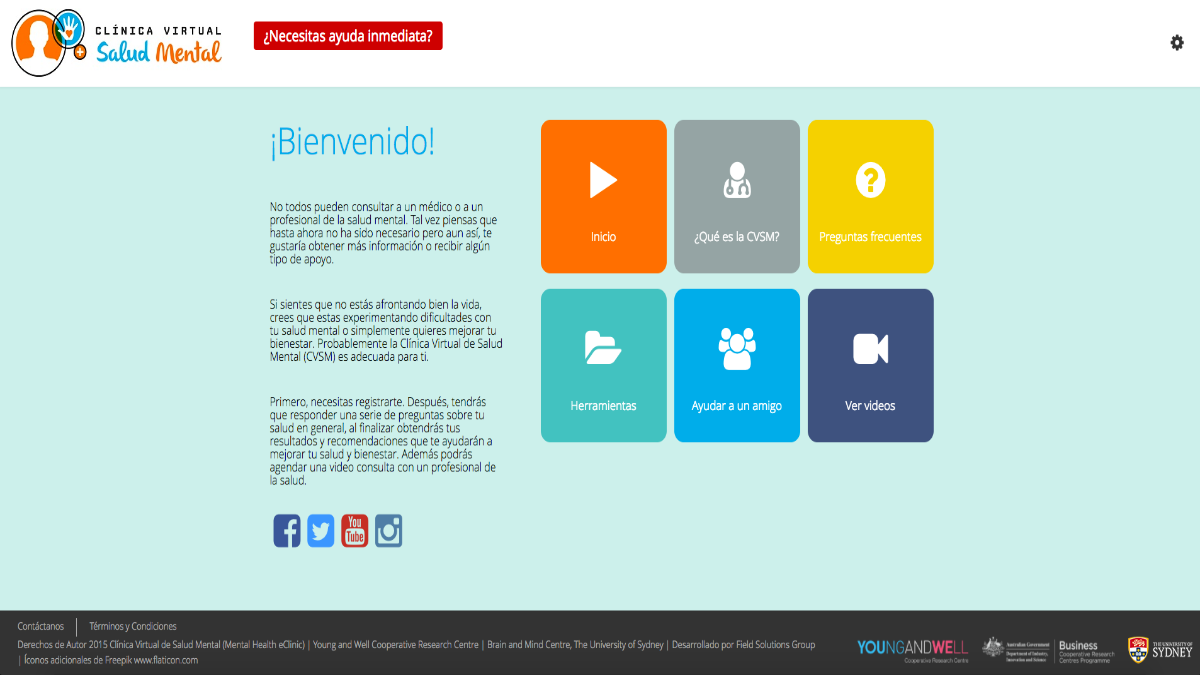
Home page and triage system.

When shown the home page, participants (15/32, 47%) suggested that the MHeC-S webpage’s domain should be “.com” or “.org,” as this would increase the website’s credibility. At the same time, they wanted the home page to display all relevant logos of affiliated organizations such as the logo of the University of Sydney and relevant Latin American or Spanish universities associated with the MHeC-S. In this space, they wanted to find a simple description of “...what the MHeC-S has to offer...” (young person, quote J) and perhaps a series of short videos that explain more about the MHeC-S and also contained testimonials. As language might be a concern, participants suggested adding a settings cog on the home page so they could choose their language and, consequently, relevant content would also be prompted. The triage system was widely accepted, as all users understood the need for screening for urgent services and for rapid referral of users (Figure 3).

##### Element 2: Online Physical and Mental Health Self-Report Assessment

**Figure 4:**
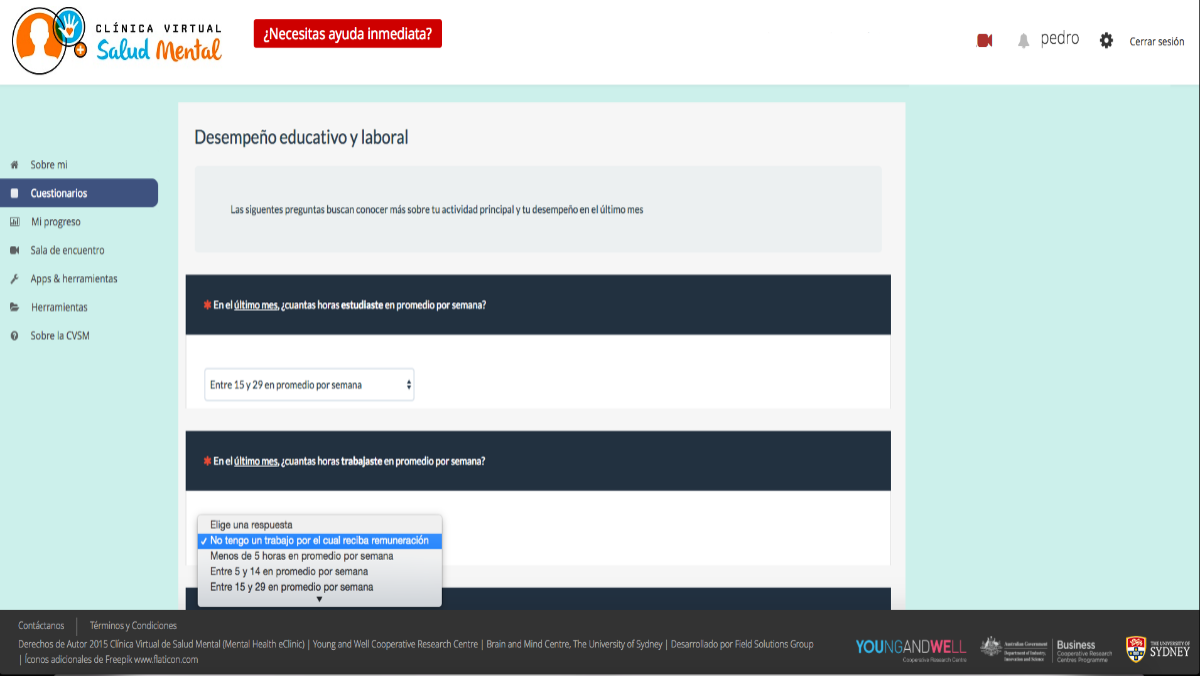
Online physical and mental health self-report assessment.

When shown the assessment (via questionnaire) (Figure 4), participants liked that the online physical and mental health assessment used rule-based decision algorithms that enable personalization of the assessment to the young person’s needs (eg, sex-specific questions or in-depth assessments according to positive screening responses) and inform the dashboard of results. They also accepted the established features of pausing and resuming later, as they would give participants more flexibility to complete the assessment where and when they prefer. Additionally, participants approved the type of questions (eg, Likert-type scale questions and 2-way closed-ended questions) contained in this assessment. However, health professionals (12/12, 100%) suggested including 1 open-text question at the beginning of the assessment with the aim of assessing the reason for accessing the MHeC-S that day, as one clinician explained:

I would like to know the reason [why the young person was] visiting the MHeC-S...as we do in practice assessing the presenting or chief complaint...

Clinician, quote K

##### Element 3: Dashboard of Results and Progress

**Figure 5:**
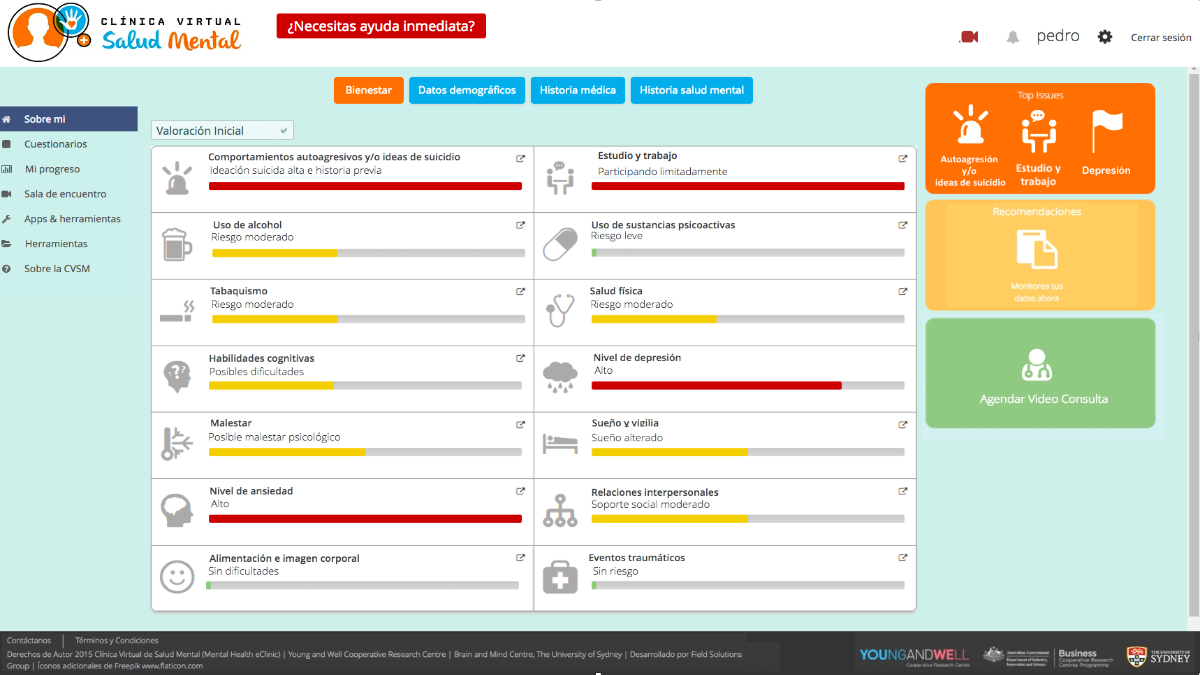
Dashboard of results and progress.

All 32 participants agreed that after completion of the online self-report assessment a dashboard of results should be displayed immediately (Figure 5). Participants accepted the simple bar and line graphs, colored icons, and traffic light representations, and reported that they were easy to understand. Health professionals (12/12, 100%) believed that the assessment and the dashboard of results were useful tools to inform their practice, not only in their first assessment but also as an ongoing form of care. In relation to the dashboard’s language, participants preferred the use of lay terms instead of medical terminology. When medical jargon is needed (eg, psychosis or hypomania), participants proposed that the prototype should display a simple explanation of the term when they click on the word or hover over it.

##### Element 4: Booking System and Video Visit System

**Figure 6:**
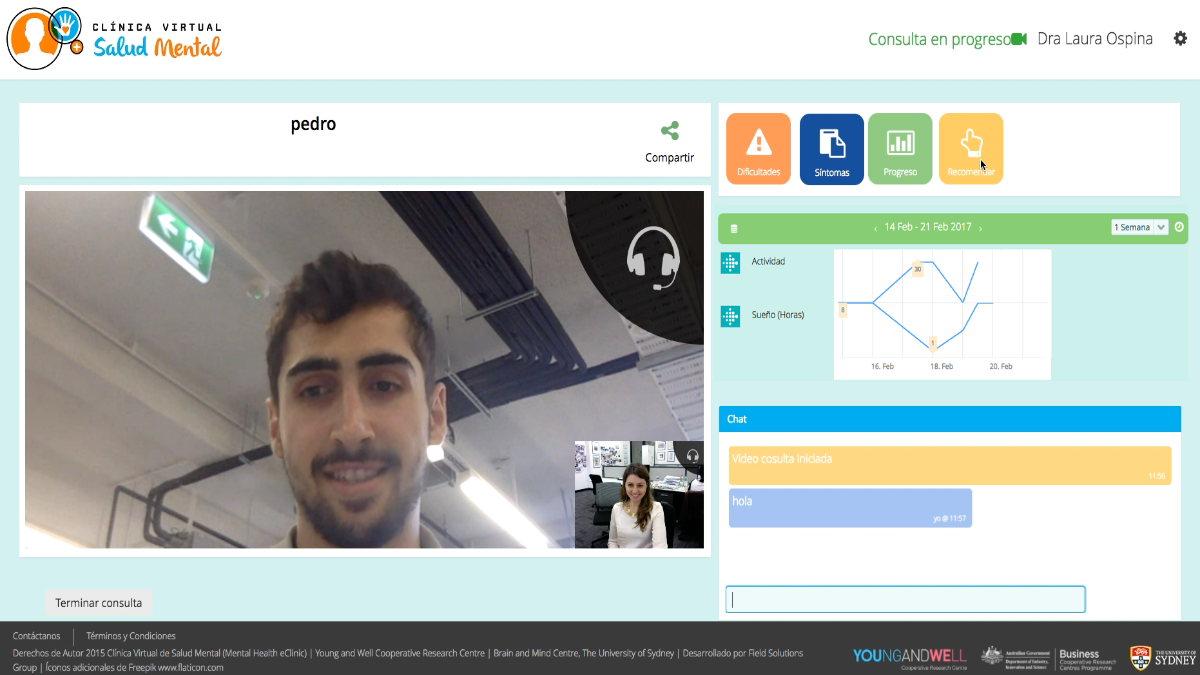
Booking system and video visit system.

All participants agreed that the booking and video visit system embedded in the MHeC-S (Figure 6) was a secure way of protecting privacy. Due to the limited numbers of Spanish-speaking health professionals in Australia, participants believed that having a video visit with a Spanish-speaking health professional would be an effective way of screening and assessment, as well as providing (and receiving) advice, treatment, and therapy. Importantly, they acknowledged that video visits would be more efficient, as this would save them time and money, as a young person explained:

...we will know exactly where to go and not to waste time going from one place to another, searching for someone that understands me...

Young person, quote L

Furthermore, the prototype provided them with security, as a health professional explained:

...they can always know where to go, like a secure base...

Health professional, quote M

##### Element 5: Personalized Well-Being Plan That Includes Links to Evidence-Based, Young Person–Suggested, Health Professional–Recommended Apps and E-Tools

**Figure 7:**
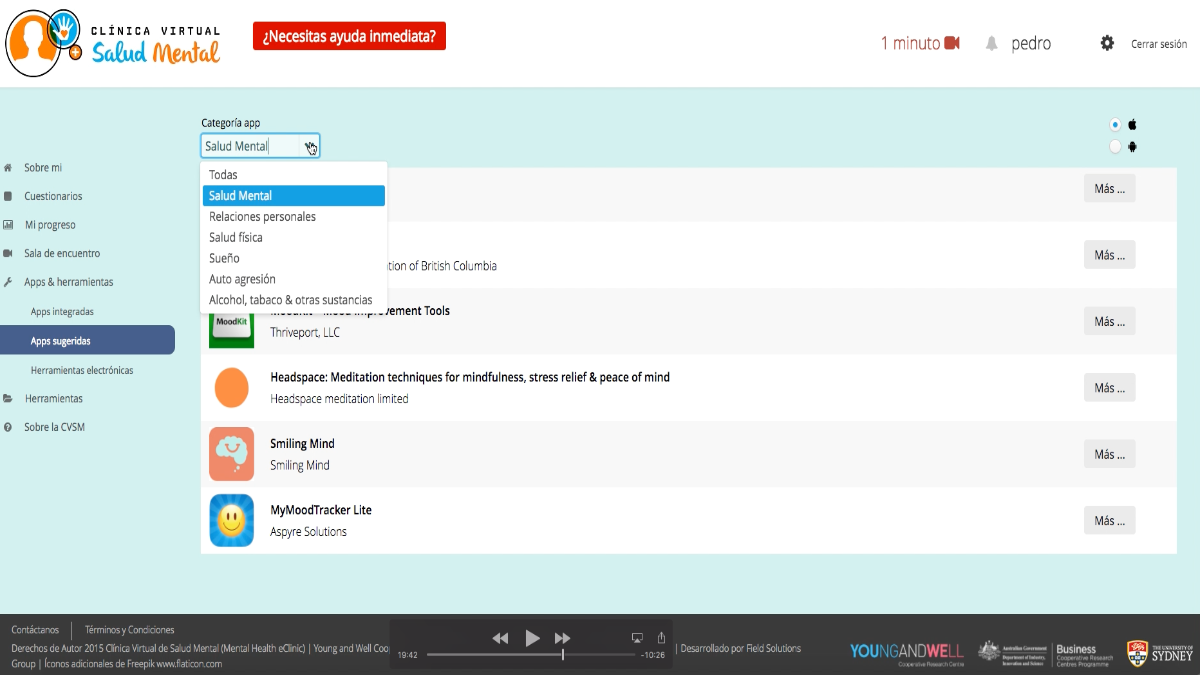
Personalized well-being plan that includes links to evidence-based, young person–suggested, health professional–recommended apps and e-tools.

The idea of having a tailored plan and recommendations immediately after the completion of the self-report assessment was widely accepted by participants (32/32, 100%) (Figure 7). Young people (17/17, 100%) said that they would be likely to download and use the recommended apps and e-tools if those matched with their needs. However, most of the participants (29/32, 91%) highlighted a lack of Spanish-language apps and e-tools . Young people (17/17, 100%) said that they would try to use an app in English, but they also recognized that their experience and the benefit would be limited, especially for those apps that have audio resources, as one young person explained:

I would try to use it as much as I can, but I think there are going to be many things I don’t understand—for example, the mindfulness audios...

Young person, quote N.

As potential solutions, participants proposed the creation of videos that contain general information, as well as relaxation and breathing exercises; a detailed directory that describes available English apps and e-tools; and, ideally, the development of several Spanish-language apps, e-tools, and Spanish adaptations of the best evidence-based resources.

#### Content: Translation and Cultural Adaptation of the Self-Report Assessment

The self-report assessment included 16 modules (Table 1 [64-89]) with smart skips built in so that it was tailored to each individual and took a minimum amount of time to complete (approximately 45 minutes).

The self-report assessment translation process started in early 2016, with the literature review. We found 8 Spanish-translated versions of measures from the original source: the 2-step method to measure transgender identity [90], 10-item Kessler Psychological Distress Scale [91], Quick Inventory of Depressive Symptomatology [92,93], Community Assessment of Psychic Experiences [94,95], Alcohol Use Disorders Identification Test [96], Alcohol, Smoking and Substance Involvement Screening Test [97], International Physical Activity Questionnaire [98,99], and the Spanish version of the World Mental Health Composite International Diagnostic Interview used in the National Comorbidity Survey Replication Adolescent Supplement [100,101]. We selected 5 because we found their translation and psychometric properties in the academic literature: the Primary Care Posttraumatic Stress Disorder (PTSD) Screen [102], PTSD Checklist-Civilian Version [103], Altman Self-Rating Mania Scale [104], the Cutting down, Annoyance by criticism, Guilty feeling, and Eye-openers questionnaire (which has been widely used in several Spanish-speaking studies and several versions are available online [105,106]; we used the Colombian version for its methods [107]), and the Fagerström Test for Nicotine Dependence [108]. Although we didn’t find any versions of the empathy quotient scale in the academic literature, we found a Spanish version provided by the Autism Research Centre of the University of Cambridge [109].

**Table 1:** Self-report assessments in each of the 19 modules.

Module		Questionnaires
1.	Main reason for visiting the MHeC-S^a^	Short open-text question
2.	General demographics	Items derived from the Second Australian Young and Well National Survey [64] and the 2-step method to measure transgender identity [65]
3.	Social and occupational function	Modified versions of the Brief Disability Questionnaire [66] and the self-report version of the Social and Occupational Functioning Assessment Scale [67]
4.	Psychological distress	10-item Kessler Psychological Distress Scale [68]
5.	Depressed mood	Quick Inventory of Depressive Symptomatology (QIDS-SR-16) [69]
6.	Anxiety	Overall Anxiety Severity and Impairment Scale [70]
7.	Mania-like experiences	Items derived from the Altman Self-Rating Mania Scale [71]
8.	Psychosis-like experiences	Items derived from the Community Assessment of Psychic Experiences-Positive Symptoms Scale [72]
9.	Traumatic experiences	Primary Care PTSD^b^ Screen [73] and the PTSD Checklist-Civilian Version [74]
10.	Self-harm behaviors and suicidal ideation	Suicidal Ideation Attributes Scale [75]
11.	Tobacco, alcohol, and substance use	Items adapted from the Alcohol Use Disorders Identification Test [76], the Alcohol, Smoking and Substance Involvement Screening Test [77], the Cutting down, Annoyance by criticism, Guilty feeling, and Eye-openers questionnaire [78], the Drinking Motives Questionnaire [79], the Fagerström Test for Nicotine Dependence [80], and selected items from the National Drug Strategy Household Survey [81]
12.	Physical activity	International Physical Activity Questionnaire [82]
13.	Sleep behaviors	Sleep-related items from the QIDS-SR-16
14.	General mental health conditions	National Comorbidity Survey Replication Adolescent Supplement [83]
15.	Overall heath and somatic distress	Somatic and Psychological Health Report [84], self-perceived health status, and general body measurements
16.	Medical, mental health, and family history	Multiple-choice questions
17.	Cognitive concerns and empathy	Derived from the Subjective Scale to Investigate Cognition in Schizophrenia [85] and the empathy quotient [86]
18.	Eating behaviors and body image	Derived from the Eating Disorder Examination [87]
19.	Social connectedness and support	Derived from the Perceived Social Support/Conflict Measure [88] plus 5 items measuring relationships with peers [89]

^a^MHeC-S: Spanish version of the Mental Health eClinic.

^b^PTSD: posttraumatic stress disorder.

We didn’t find any Spanish versions of the items assessing disability, suicide ideation, and anxiety rating. Considering their relevance in overall assessment and potential medicolegal repercussions, we decided to find Spanish-speaking analogs to these measures. We replaced the Brief Disability Questionnaire with the World Health Organization Disability Assessment Schedule 2.0, which has an official translation available [110]. We replaced the Suicidal Ideation Attributes Scale with the Suicide Behaviors Questionnaire-Revised [111] and we replaced the Overall Anxiety Severity and Impairment Scale with the 7-item Generalized Anxiety Disorder scale [112], both of which have their translation process and psychometric properties published.

In July 2016, the rest of the items were carefully translated into 3 individual sessions of colloquial Spanish, and we conducted 1 round of translation and cultural adaptation for Spanish-speaking populations living in Australia. At this stage, we adapted 3 questions in the demographics sections: country of origin; language spoken at home, enriched with relevant dialects from Spanish-speaking regions, such as Quichuan and Catalan; and the ethnicity question, modified to the indigenous populations in Latin America.

To reach agreement, 2 individual sessions of back-translations were performed, followed by 1 round of discussion between the translators and back-translators.

#### User Interface

**Figure 8:**
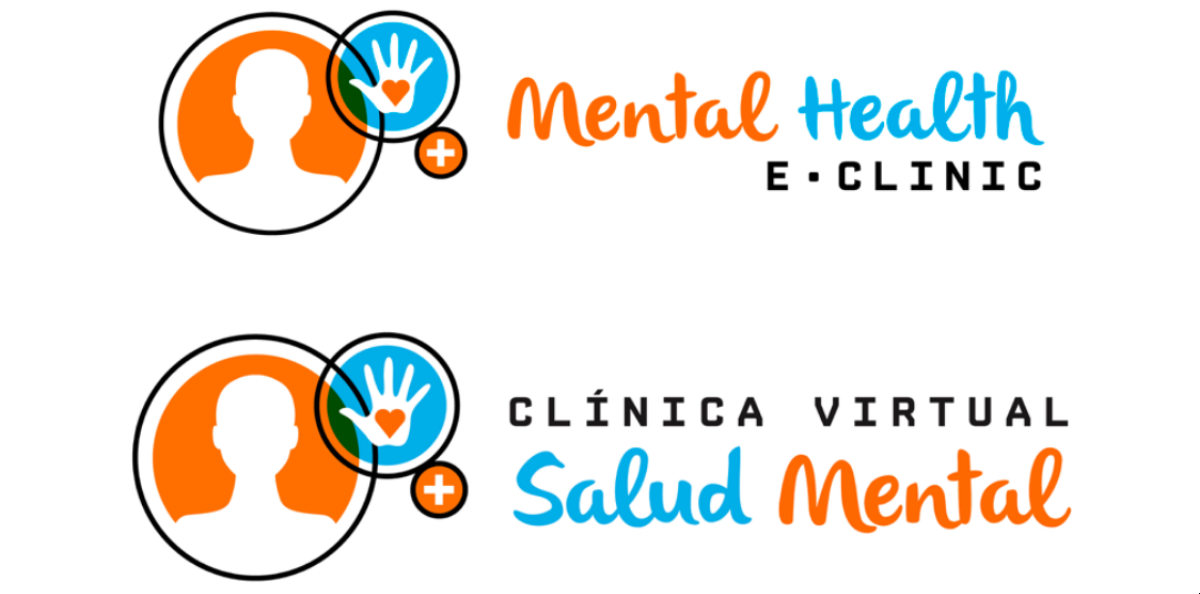
Original Mental Health eClinic logo and its Spanish adaptation. Created by Mandarin Creative

All participants accepted the Spanish version of the original MHeC’s logo (Figure 8). However, young people preferred a name that they could associate more with general well-being than with mental health, as some of them believed that this would have a wider reach among students.

In relation to language, participants expressed different preferences for the linguistic form in which to address the users (interlocutors); Colombian participants (20/32, 63%) favored the use of formal pronouns (usted), as they considered that the delivery of online health services should follow the same conventions as face-to-face services:

In Colombia, the doctor-patient relationship is always treated in a formal way...

Health professional, quote O

Participants of other nationalities (12/32, 38%) preferred the prototype to use the colloquial or familiar pronouns (tú, vos), as the formal pronoun seemed excessively formal in an online context. Considering this discrepancy, all participants agreed that the prototype would use the colloquial or familiar form of the second person singular pronoun (tú), as the target of the MHeC-S is young people from different nationalities. Additionally, participants suggested the possibility of a customizing option to choose to see the prototype (1) completely in Spanish (including menus, links, call-to-action buttons, instructions, videos, apps, and e-tools), (2) in a bilingual version (which would display menus, call-to-action buttons, and instructions in English, but the most relevant content in Spanish, such as the physical and mental health self-report assessment and video visit; or a mix of Spanish and English apps, e-tools, and resources), or (3) completely in English (which would look more like the original MHeC but with relevant information for this population).

Participants in the one-on-one user testing sessions (n=15) assessed the interface in the prototype. These participants approved the MHeC-S’s font, color palette (light blue, orange, and green), and the tile-shaped buttons (15/15, 100%). Despite this, young people (7/15, 47%) thought that the Get Started call-to-action button needed to be different (bolder, bigger, brighter, or in a different shape) to get the participants to sign up. In relation to the menus, horizontal or hamburger displays were preferred over the current vertical presentation.

#### Usability

A total of 15 participants completed 4 usability tasks: (1) create an account and log in, (2) find the Need Help Now button, (3) explore the dashboard of results, and (4) book an appointment. Mean SEQ scores for the tasks were 7 (SD 0); 6.93 (SD 0.26); 5.07 (SD 1.49); 5.80 (1.66) respectively, range 1 to 7. All 15 participants did not report problems completing tasks 1 and 2. In relation to task 3, participants said that exploring all of the dashboard tabs was slightly complicated, as they were not evident at first glance. They had slight difficulty in completing an appointment booking, as the action button was located on the bottom right corner of the site, so this task wasn’t intuitive for some participants.

## Discussion

### Principal Findings

Our study used a comprehensive research and development approach to co-design and culturally adapt a prototypic Web-based mental health clinic (MHeC) for Spanish-speaking young people based in Australia (MHeC-S). Thematic analysis resulted in adequate acceptability of the 5 main elements of the alpha prototype (a home page and triage system; a comprehensive online physical and mental health self-report assessment; a dashboard of results and progress report; a booking and videoconferencing system to enable video visits; and the generation of a personalized well-being plan that includes links to evidence-based, young person–suggested, health professional–recommended apps and e-tools). The data also revealed gaps in the alpha prototype, such as the need for tailored assessment tools and a greater integration with Spanish-speaking services and communities; a lack of Spanish-language apps and e-tools, and of online mental health information was noted. As a consequence, the development of new features included the addition of cultural adjustment items in the online self-report assessment, creation of specific algorithms, and development of several videos and factsheets (Multimedia Appendix 2). In the future, the beta prototype should additionally include refinements of language; explanations of specific medical terminology; and minor changes in layout and navigation.

Migrants and newly arrived residents have been identified as populations who are difficult to recruit, and then to involve and maintain in research [113], yet this is a population in critical need of support. The research and development cycle that we employed in this study is an optimal methodology to engage, retain, and work more efficiently with hard-to-reach populations. We selected various participatory design methodologies to enhance the generation of new ideas and improve the feedback process. The nature of the research and development cycle and the use of diverse methodologies enabled the research to be conducted and completed in a time-efficient manner.

Previous research has highlighted the need to tailor HIT interventions beyond content and language, by including culture [39]. One of the strengths of this study was the incorporation of the cultural framework as a cornerstone of the research and development cycle. As a consequence, we obtained information about the participants’ cultural preferences for the prototype’s interface and functionality, as well as the development of culturally appropriate content and features. Performing data collection and analysis in the original language reduced the risk of losing relevant information (or meaning), and decreased research time and costs [114]. Other advantages of this study were the variety of origin of participants (Argentina, Chile, Colombia, Spain, and Venezuela) and the research team (Australia, Chile, Colombia, and Venezuela). Furthermore, this research united all relevant stakeholders (young people, supportive others, and health professionals) in a common goal of adapting this prototype to a population in need.

Although Spanish is the second most common language spoken worldwide and HIT is a growing field, Spanish-speaking populations (including migrants and those residing in low- and middle-income countries) are at risk of experiencing not only physical but also technological social health inequalities [115]. This body of research aims to breach this gap by creating a widely available MHeC-S that works across devices. The participation of end users in the design process ensured that the prototype was accessible to individuals of varying literacy levels with a range of cultural differences. Furthermore, the MHeC-S has the potential to be configured and adapted for use in Spanish-speaking countries and in other multicultural countries with Spanish-speaking migrant populations.

### Implications

International education in Australia has grown dramatically and is its third largest export industry, contributing Aus $32.4 billion to the Australian economy [116]. It highlights a significant bilateral exchange (Aus $755 million in 2012) between Latin America and Australia, which is increasingly recognized as an important destination for the English education of Latin Americans [117]. In 2017, the Latin American Spanish-speaking international student population had reportedly increased to more than 21,000 [17]. Our study highlighted a critical concern in the community in relation to a shortage of mental health services targeting the well-being of these students. This is vital, as these students are at higher risk of developing adaptational problems and being socially and linguistically isolated [118]. Participants generally expressed a lack of understanding of the Australian health system, particularly service providers and insurance agencies, resulting in an important barrier for students’ help-seeking process. Even for those who do know how to navigate the health system, a reduced English competence could impair the care they do obtain. Protecting, caring for, and enhancing positive experiences for international students is Australia’s best strategy to protect and grow this industry.

New and emerging technologies present a solution, as they have changed the way young people communicate, connect, and engage with each other and with society. With the introduction of smartphones, information, services, and resources provided online or via mobile apps can be accessed privately and at any time. This can be empowering for individuals who are marginalized or geographically or socially isolated. It could also help to address the need for Spanish-speaking mental health professionals and interpreters. Having an MHeC-S could greatly benefit young people who are native Spanish speakers living in Australia and who are actively seeking help. This study is the first step toward providing a technology-enabled solution to improve this population’s mental health and well-being in Australia. To the best of our knowledge, there has been no research to date in this field.

### Conclusion

Further research is needed to understand the psychometric properties of the online self-report assessment (eg, criterion validity) and the integration of the MHeC-S with other apps or e-tools. Importantly, additional steps are needed to evaluate the engagement, efficacy, and effectiveness of the MHeC-S in real-world settings. The MHeC-S shares the same elements and functionality of the original version of the MHeC. Its main difference relies on interaction with face-to-face services. The original MHeC was designed to work with primary care mental health services; however, in the case of the MHeC-S, in Australia it could be used additionally by language schools and Overseas Student Health Cover providers. To the best of our knowledge, this study is the first to explore mental health care barriers and facilitators, and potential technology solutions in a language student population; additional research is needed to expand the knowledge on this topic.

## Appendix

Multimedia Appendix 1 Original quotes in Spanish.

Multimedia Appendix 2 Development of new features during the rapid prototyping phase.

## Abbreviations

HIT: health information technology
MHeC: Mental Health eClinic
MHeC-S: Spanish version of the Mental Health eClinic
NSW: New South Wales
PTSD: posttraumatic stress disorder
SEQ: Single Ease Question
