# Correction: Using Participatory Design Methodologies to Co-Design and Culturally Adapt the Spanish Version of the Mental Health eClinic: Qualitative Study

**DOI:** 10.2196/37679

**Published:** 2022-03-03

**Authors:** Laura Ospina-Pinillos, Tracey Davenport, Antonio Mendoza Diaz, Alvaro Navarro-Mancilla, Elizabeth M Scott, Ian B Hickie

**Affiliations:** 1 Brain and Mind Centre The University of Sydney Sydney, NSW Australia; 2 Department of Psychiatry and Mental Health Pontifical Javeriana University Bogota Colombia; 3 School of Psychiatry Department of Medicine University of New South Wales Sydney, NSW Australia; 4 Neuropsychiatry Research Group Autonomous University of Bucaramanga Bucaramanga Colombia; 5 School of Medicine University of Notre Dame Australia Sydney, NSW Australia

In “Using Participatory Design Methodologies to Co-Design and Culturally Adapt the Spanish Version of the Mental Health eClinic: Qualitative Study” (J Med Internet Res 2019;21(8):e14127), the authors noted one error.

In the originally published article, a value appeared incorrectly in the following sentence.

A total of 7 health professionals participated in the workshops; 6 were female and their ages ranged from 22 to 24 years (median age 28 years).

This has been corrected as follows:

A total of 7 health professionals participated in the workshops; 6 were female and their ages ranged from 22 to 34 years (median age 28 years).

The correction will appear in the online version of the paper on the JMIR Publications website on March 4, 2022, together with the publication of this correction notice. Because this was made after submission to PubMed, PubMed Central, and other full-text repositories, the corrected article has also been resubmitted to those repositories.

