# Supplementary material for: Using Participatory Design Methodologies to Co-Design and Culturally Adapt the Spanish Version of the Mental Health eClinic: Qualitative Study
Source: J Med Internet Res. 2019 Aug 2;21(8):e14127. doi: 10.2196/14127 (PMC6696860; doi:10.2196/14127)
Supplement: Multimedia Appendix 1 [file jmir_v21i8e14127_app1.pdf]

Multimedia Appendix 1

Quotes in Spanish

A. *…even if I needed to call 000, I wouldn't be sure if they understand what I'm saying…:*
Aun si necesitara llamar al 000 no estaría seguro que me entenderían lo que digo

B. *I don't think that I would be able to explain my feelings to someone in English...:*
No creo que sea capaz de explicar a alguien mis sentimientos en inglés

C. *…it's very hard to arrive here (Australia) and not understand what is happening…*:
Es muy difícil llegar acá (Australia) y no entender nada de lo que esta pasando

D. *…understanding simple instructions – like where is the train stop – is very difficult…*:
Entender instrucciones simples cómo, donde queda la parada del tren, es difícil

E. *…it's like in English I'm a different person; sometimes I feel people think I'm dumb…:*
Es cómo si en ingles fuera una persona y en español otra, a veces creo que la gente piensa que soy tonto.

F. *…the impact on their [international students] confidence is huge. Sometimes I have to remind him [international student] what he is capable of …*:
El impacto en su confianza es gigante (estudiantes internacionales), muchas veces me toca sentarme a recordarles de las cosas que son capaces.

G. *…it is hard to try to fit, and try to understand how things work here…*:
Es difícil encajar y además entender cómo funcionan las cosas acá

H. *…the majority [of] us have Bachelor degrees in our home countries...so when we arrive in Australia, the jobs we find are very different from what we have been trained in—most of us have to work cleaning, or as a waitress or in construction…*:
La mayoría somos profesionales en nuestros países, sin embargo cuando llegamos a Australia nos toca trabajar en cualquier cosa, muchos trabajamos en limpieza como meseros o en construcción

I. *…possibly one international student dies by suicide every year here; and, more frequently we have to provide assistance to students that are hospitalized for a mental health concern...:*
Hay posiblemente un suicidio cada año de un estudiante internacional y cada vez son más y más los casos en los que tenemos que prestar asistencia por hospitalizaciones relacionadas a enfermedades mentales

J. *"…what the MheC-S has to offer…"*:
Qué me ofrece la clínica virtual

K. *I would like to know the reason [why the young person was] visiting the MHeC-S...as we do in practice assessing the presenting or chief complaint ….:*
Me gustaría saber la razón por la cual acceden al sistema ese día, tal cual lo hacemos en la practica clínica cuando preguntamos el motivo de consulta.

L. *…we will know exactly where to go and not to waste time going from one place to another, searching for someone that understands me...*:
Sabríamos exactamente a donde tenemos que ir para encontrar a alguien que nos entendiera y no perderíamos tiempo yendo de un sitio a otro.

M. *…they can always know where to go, like a secure base...*:
Ellos pueden saber a donde ir en cualquier momento, como tener una base segura.

N. *I would try to use it as much as I can, but I think there are going to be many things I don't understand—for example, the mindfulness audios …*:

Yo las intentaría usar, pero sé que hay muchas cosas que no voy a entender, por ejemplo: en las apps que tienen audios de mindfulness

O. *In Colombia, the doctor-patient relationship is always treated in a formal way...*: En Colombia, la relación medico-paciente es muy formal, se habla de usted
