# Supplementary material for: Using Participatory Design Methodologies to Co-Design and Culturally Adapt the Spanish Version of the Mental Health eClinic: Qualitative Study
Source: J Med Internet Res. 2019 Aug 2;21(8):e14127. doi: 10.2196/14127 (PMC6696860; doi:10.2196/14127)
Supplement: Multimedia Appendix 2 [file jmir_v21i8e14127_app2.pdf]

Multimedia Appendix 2

Development of new features

Participant feedback also lead to the development of new features during the rapid prototyping phase:

1) Videos: a total of four Spanish videos were filmed, the first one was an introductory video located in the home page, in which LOP describes the MHeC-S, it's utility, and how to use it. The other three videos are located in the recommended apps and etools sections. In these videos a young person presents three different relaxation and breathing exercises. Additionally, the team added Spanish subtitles to three previously created MHeC promotional videos, which targeted a general audience and contain information about: the development of the prototypic version of the MHeC; case examples; and the potential utility of the clinic in different settings including LMICs.

2) Factsheets: Eight factsheets were translated and adapted into Spanish including: how to improve sleep patterns; help with suicidal thoughts and self-harm behaviors; reduction of tobacco, alcohol and other substances; stress reduction strategies; problem solving guidelines; how to improve physical health; online help for depression and anxiety; and how to track data using mobile devices. Moreover, an additional factsheet was created, explaining how help can be sought. This document contained detailed information on where to get urgent help (000 service); immediate psychological attention (i.e helplines including: Lifeline, Kids Help Line, Suicide Call Back Service, and beyondblue); medical attention; and information about OSHC and the major transcultural mental health services. Importantly, this factsheet contained information of how to access these services in Spanish through the TIS or the service specific interpreting service.

3) Questionnaires: Assessing cultural adaptation and adjustment disorder was an essential feature in order to respond to the end users' needs. After the literature review process, the consensus identified the Adaptation Scale (BSAS), the Brief Psychological, Adaptation Scale (BPAS), the Brief Perceived Cultural Distance Scale (BPCDS) and the Brief Acculturation Orientation Scale (BAOS) [118] as the most appropriate questionnaires to be incorporated to the online assessment as they measure all the acculturation spectrum, have good psychometric properties and has been translated in nine languages including Spanish.

4) Algorithms: in order to tailor the online physical and mental health assessment to the IntSt population the questionnaire-based cutoffs were modified to reflect the Spanish-language validated questionnaires cutoffs. Moreover, a total of eight new algorithms were created in order to reflect the questionnaires added to the online physical and mental health assessment (BDQ, SBQ-R, GAD-7, BSAS, BPAS, BPCDS, BAOS and the dialect question). Finally, for those who scored at high risk while completing the SBQ-R an "emergency" algorithm was created. This means, if a young person is identified as being at significant risk of harm (self-harm or suicidal ideation) while completing the online physical and mental health assessment, the MHeC-S will immediately provide the emergency contact details and will encourage the individual to seek help from their local general practitioner. Additionally, the MHeC-S offers specific advice (via factsheet) as to how to access appropriate counselling, support or formal health care.
